# PSA Density and PIRADS 5 Lesions as Key Determinants of Upstaging After Radical Prostatectomy

**DOI:** 10.3390/cancers18081319

**Published:** 2026-04-21

**Authors:** Patryk Patrzałek, Mikołaj Kisiała, Marcel Dawidowicz, Jakub Wieland, Karol Zagórski, Jakub Karwacki, Adam Gurwin, Jan Łaszkiewicz, Wojciech Tomczak, Wojciech Urbański, Dawid Janczak, Wojciech Krajewski, Tomasz Szydełko, Bartosz Małkiewicz

**Affiliations:** 1 University Center of Excellence in Urology, Department of Minimally Invasive and Robotic Urology, Wroclaw Medical University, 50-556 Wroclaw, Poland; 2 University Center of Excellence in Urology, Wroclaw Medical University, 50-556 Wroclaw, Poland; 3 Department of Pathophysiology, Wroclaw Medical University, 50-368 Wroclaw, Poland; 4 Clinical Department of Rheumatology and Internal Diseases, Institute of Internal Diseases, Wroclaw Medical University, 50-556 Wroclaw, Poland

**Keywords:** prostate cancer, pathological upstaging, radical prostatectomy, risk prediction, clinical staging, multiparametric MRI, fusion biopsy

## Abstract

**Introduction**: Clinical staging based on digital rectal examination is imprecise, leading to pathological upstaging in patients with prostate cancer (PCa). Accurate preoperative assessment remains a challenge despite the use of multiparametric magnetic resonance imaging (mpMRI) and fusion-guided biopsy. This study aims to identify key predictors of upstaging in preoperative patients. **Materials and Methods**: A retrospective analysis of 924 patients who underwent radical prostatectomy between July 2012 and January 2025 was performed. Variables included prostate-specific antigen, prostate volume, biopsy type, MRI, body mass index and age. Upstaging was defined as ≥pT3 in patients staged clinically as cT1–2. Optimal cut-offs for continuous variables were defined statistically. Multivariable logistic regression was applied to identify independent predictors of upstaging and minor staging upgrading (MSU)—defined as any upward shift in the pathological T stage relative to the clinical T stage. Model performance was evaluated using the area under the Receiver Operating Characteristic (ROC) curve (AUC). **Results**: Upstaging occurred in 31.9% and MSU in 50.6% of patients. The mean age was 65 years. Cut-off values for PSA density (PSAD) were 0.29 for upstaging and 0.28 for MSU. In the full-cohort model (AUC = 0.628), PSAD (odds ratio (OR) = 2.55), age (OR = 1.04), and hypertension (HT) (OR = 1.47) were associated with upstaging. In PIRADS-based models, PIRADS 5 and PSAD predicted both upstaging (OR = 1.62 and 6.10, respectively; AUC = 0.664) and MSU (OR = 1.75 and 4.67, respectively; AUC = 0.659). MSU was also associated with HT and a lack of fusion biopsy (AUC = 0.622). **Conclusions**: PSAD and PIRADS 5 lesions are strong determinants of pathological upstaging and MSU in PCa. These factors should be considered in preoperative risk stratification to improve staging accuracy. Despite advances in imaging and biopsy techniques, upstaging remains a common phenomenon, underlining the need for further refinement of diagnostic protocols.

## Simple Summary

Many patients with prostate cancer appear to have disease confined to the prostate before surgery, but the final pathology report may reveal more advanced cancer than initially expected. This discrepancy can influence treatment planning, including decisions regarding surgery, active surveillance, and postoperative management. We retrospectively analyzed a large cohort of men who underwent prostate removal surgery to identify which preoperative factors were associated with occult advanced disease. We found that a higher prostate-specific antigen level in relation to prostate size, as well as highly suspicious findings on magnetic resonance imaging, were the strongest indicators of underappreciated disease extent. Older age and high blood pressure were also associated with an increased risk in some analyses. These findings may help clinicians better assess cancer stage before treatment and support future research aimed at developing more accurate prediction tools for prostate cancer management.

## 1. Introduction

The European Association of Urology (EAU) recommended clinical T staging in prostate cancer (PCa), which relies on digital rectal examination (DRE) [1]. However, DRE has limited diagnostic accuracy [2]. Subjective examination frequently misrepresents tumor stage, leading to discrepancies with the final pathology and potentially compromising optimal treatment selection. Upgrading (defined as an increase in Gleason Grade Group between biopsy and final pathology) and upstaging (defined as pathological T stage ≥ pT3 despite lower clinical stage) are significant clinical challenges. Their frequency is critical for optimizing care and individualizing treatment [3].

Accurate staging improves outcomes and reduces recurrence and mortality. Notably, patients who experience upgrading or upstaging have a 1.5-fold increased risk of positive surgical margin (PSM) on radical prostatectomy (RP) pathology. Moreover, both upgrading and upstaging are associated with a 1.8- and 2.1-fold risk of BCR, respectively [4]. Discrepancies in staging and grading may lead to significant clinical consequences, including a shift from active surveillance to radical surgical intervention [5]. Moreover, accurate staging enables tailored treatment and avoids undertreatment [6].

Modern preoperative techniques include multiparametric MRI (mpMRI) and MRI-TRUS fusion biopsy, which are now the gold standard in treatment planning for patients with PCa, as recommended by the EAU Guidelines [1]. These methods reduce the risk of staging errors [7]. Modern imaging, combined with conventional diagnostic parameters such as PSA levels and PSA density (PSAD) and clinical data, provides a comprehensive and individualized risk assessment of cancer progression. Despite its value, staging remains suboptimal.

The aim of this study is to develop a multivariable model for predicting pathological upstaging in patients with PCa based on preoperative clinical, imaging, and biopsy data. By analyzing a large, well-characterized cohort of patients undergoing RP, the study also quantifies the extent of preoperative disease underestimation. Furthermore, it assesses the role of key clinical and diagnostic variables in enhancing risk stratification and informing treatment decisions.

## 2. Materials and Methods

### 2.1. Study Population

We conducted a retrospective single-center analysis at the University Centre of Excellence in Urology, Wroclaw Medical University, Poland, on a cohort of 924 patients who underwent RP between July 2012 and January 2025. The study was approved by the institutional bioethics committee (approval number: KB 755/2022). Out of the initially screened 1343 patients, 419 were excluded due to missing key clinical or pathological data. The inclusion criteria were the availability of clinical staging (cT), histopathological staging (pT), and detailed preoperative biopsy data. In the analyzed cohort, no patients had received neoadjuvant chemotherapy, preoperative hormone therapy, or preoperative radiotherapy.

### 2.2. Clinical Variables

Studied parameters included PSA concentration [ng/mL], prostate volume [mL], PSAD, [ng/mL/cm^3^], PIRADS 5 lesion presence (in cases where mpMRI were available), Body mass index (BMI), age, hypertension (HT), and diabetes mellitus, and whether a fusion biopsy was performed. cT was determined following the EAU guidelines. Prostate biopsies were performed within the institutional diagnostic pathway according to standardized procedural protocols. However, operator-related differences in sampling technique may occur, and cannot be fully excluded in this retrospective study.

### 2.3. Surgical Approach

The surgical approach was not an inclusion or exclusion criterion; therefore, the cohort comprises patients who underwent open (*n* = 473), laparoscopic (*n* = 194), or robot-assisted radical prostatectomy (RARP) (*n* = 257). Postoperative pT was determined according to standard pathological evaluation protocols.

### 2.4. Radiological Evaluation

mpMRI imaging examinations were interpreted using standardized radiological reporting protocols applied at our institution throughout the study period. Each MRI report was evaluated by at least one radiologist with a minimum of 6 years of experience in uropathology imaging. Although interobserver variability in MRI interpretation could have influenced lesion classification, the use of structured reporting and experienced readers was intended to reduce such variability. Nevertheless, operator-dependent effects cannot be fully excluded in this retrospective study.

### 2.5. Definitions of Upstaging and MSU

Both upstaging and minor staging upgrading (MSU) were evaluated in the analyzed cohort. Upstaging was defined as a pathological diagnosis of advanced disease (≥pT3) that was not clinically suspected preoperatively. MSU referred to any upward shift in pT relative to cT, excluding changes that fulfilled the definition of upstaging. To ensure consistency in staging comparisons, the cT1 category, which lacks a direct pathological counterpart since pT1 is not defined, was standardized as follows: clinical T1 cases (e.g., T1c) were treated as equivalent to cT2a for comparative analysis. This assumption was necessary to enable symmetric comparison between clinical and pathological staging systems, as direct cT1-to-pT mapping would otherwise introduce inherent classification asymmetry. From a clinical perspective, cT1c tumors represent non-palpable, that is typically organ-confined, and therefore most closely corresponds to the lowest pathological category of localized disease (pT2a). Importantly, without such standardization, any transition from cT1c to pT2 would be systematically classified as upgrading, leading to an artificial inflation of MSU rates driven by structural limitations of the TNM classification rather than true biological discordance. Accordingly, a transition from cT1c to pT2a was not classified as MSU or upstaging. In contrast, a shift from cT1c/cT2a to pT2b/pT2c was considered a minor shift but not an upstaging. This approach was adopted to avoid artificial staging discrepancies caused by classification limitations while preserving methodological consistency.

### 2.6. Statistical Analysis

#### 2.6.1. Distribution Testing and Group Comparisons

The normality of the distribution of continuous variables was assessed using the Shapiro–Wilk test. In cases where the test indicated a non-normal distribution, non-parametric tests were applied accordingly. Comparisons between groups were performed using the Mann–Whitney U test for quantitative variables and the chi-square or Fisher’s exact test for qualitative variables.

#### 2.6.2. ROC Curve and Cut-Off Point Analysis

Receiver Operating Characteristic (ROC) curve analyses were performed for selected continuous variables to evaluate their prognostic value. The predictive accuracy of single determinants was also evaluated using the area under the ROC curve (AUC). The Youden index was applied to determine optimal cut-off points for each variable, representing the threshold with the best balance between sensitivity and specificity.

#### 2.6.3. Logistic Regression Models

Multivariable logistic regression models were used to identify independent risk factors for upstaging and MSU. Four models were evaluated: upstaging the entire cohort (*n* = 924), upstaging the cohort including PIRADS (*n* = 326), the MSU model for the entire cohort (*n* = 924), and the MSU model including PIRADS (only patients with a PIRADS score; *n* = 326). Prior to model construction, collinearity among explanatory variables was assessed using the variance inflation factor, and interaction terms were tested to evaluate potential effect modification. Predictive accuracy was assessed using the AUC.

All candidate variables were initially evaluated in univariable logistic regression analysis. Variables with a *p*-value < 0.05 were subsequently included in multivariable models, provided that no significant collinearity was identified. This approach was applied consistently across both upstaging and MSU analyses to ensure methodological coherence and to reduce the risk of overfitting while retaining clinically relevant predictors.

The multivariable models for upstaging were constructed based on clinical hypotheses and prior evidence from the literature, allowing the inclusion of variables considered clinically relevant regardless of their statistical significance in univariable logistic regression. In contrast, the MSU models were designed as an exploratory analysis. Consequently, to minimize the risk of overfitting, the multivariable model for MSU included only those variables that demonstrated a *p* < 0.1 in univariable logistic regression analysis.

The study aimed to develop a prognostic tool based on preoperative data to predict the likelihood of upstaging. The model’s predictive performance was evaluated using ROC curve analysis, and the cut-off value was determined using the Youden index as the probability threshold that provided the best balance between sensitivity and specificity. The odds ratio (OR) for a 0.1-unit increase in PSAD was calculated using the formula OR = eβ⋅ΔxOR = eβ⋅Δx, which represents a rescaling of the logistic regression coefficient for continuous predictors. This method allows for a more clinically meaningful interpretation of the effect size associated with incremental changes in continuous variables.

#### 2.6.4. Software

All analyses were performed using Statistica software, version 13.3.721.1.

## 3. Results

Upstaging was confirmed in 295 (31.9%) patients, while MSU was observed in 468 (50.6%) patients among the 924 men who underwent RP. The mean age of the entire cohort was 65.0 ± 6.2 years. Mean preoperative PSA concentration, PSAD, and mean prostate volume were 13.2 ± 12.8 ng/mL, 0.2 ng/mL/cm^3^, and 42.0 ± 19.0 mL, respectively. Fusion biopsy was performed in 224 (20%) patients, and 326 (35.3%) men underwent MRI, which was available for further analysis. Detailed data are presented in Table 1.

**Figure 1:**
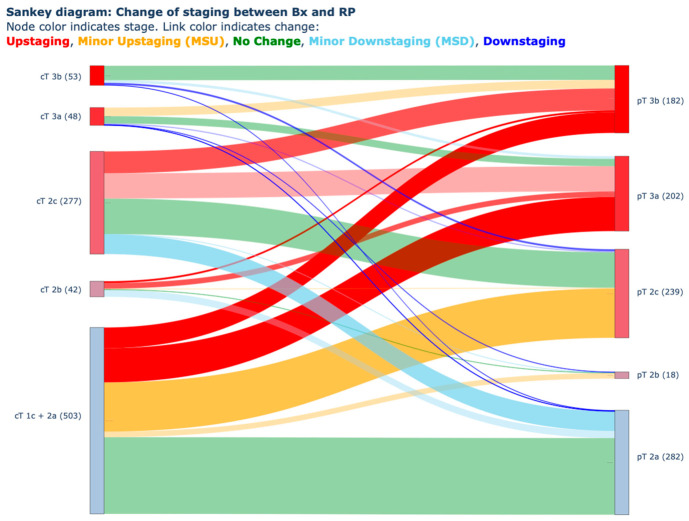
Sankey diagram illustrating changes in clinical stage between initial biopsy and final radical prostatectomy (RP) pathology. Flow thickness corresponds to the number of patients transitioning between categories. Legend: Node color indicates stage (1 = blue-ish, 5 = red). Link color indicates change (red = Upstaging, blue = Downstaging, green = no change, yellow = Minor Upstaging (MSU), azure = Minor Downstaging (MSD)).

A comparison between patients with and without upstaging revealed several significant differences. Upstaging occurred in 295 vs. 629 patients. The upstaging group was on average 1.4 years older (65.9 vs. 64.5 years; *p* = 0.003) and had a higher prevalence of HT (52.5% vs. 44.3%; *p* = 0.019). PSA levels were higher (15.1 vs. 12.4 ng/mL; *p* = 0.000002), prostate volume was smaller (39.5 vs. 43.1 mL; *p* = 0.0041), and PSAD was greater (0.429 vs. 0.319 ng/mL/cm^3^; *p* = 0.00001). Upgrading was also more frequent in this group (54.8% vs. 47.8%; *p* = 0.049). The exact staging changes, along with their proportions, are presented in Figure 1.

To increase utility, the AUC was calculated to assess the predictive performance of selected continuous variables. The AUC for age and PSAD was 0.57 and 0.62, respectively. The optimal cut-off values, as determined by the Youden index, were 68 years for age and 0.29 ng/mL/cm^3^ for PSAD.

### 3.1. Upstaging—Entire Cohort

In the full-cohort model including PSAD, age, HT, BMI, and type 2 diabetes, only PSAD, age and HT were significantly associated with upstaging. PSAD had the most substantial effect (OR = 2.56; 95% CI: 1.71–3.84; *p* < 0.001); each 0.1-unit increase raised the odds by 9.8% (95% CI: 5.5–14.4%). Age was also predictive, with each additional year increasing the odds by 4.2%. HT was a positive indicator, while BMI and diabetes were not significant. Full results are shown in Supplementary Material S1.

The model’s AUC was 0.628, indicating moderate discrimination. Although overall performance was limited (AUC 0.6–0.7), PSAD may be useful in identifying patients at risk of upstaging, pending external validation.

### 3.2. Upstaging MRI Model

**Figure 2:**
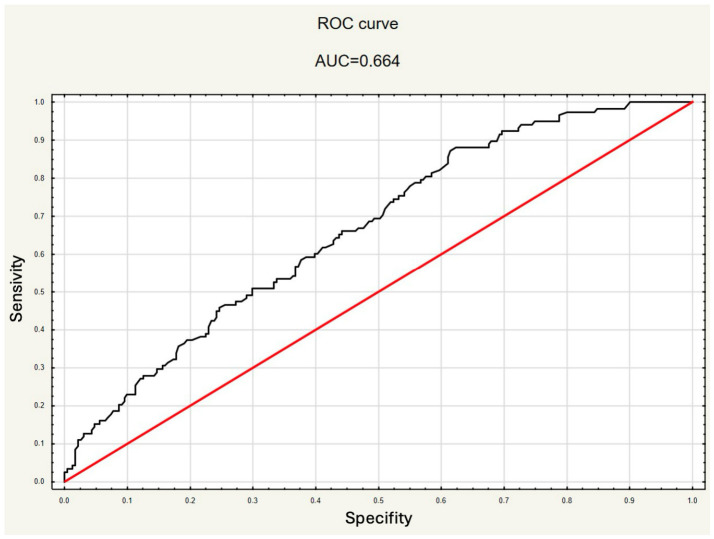
ROC Curve for Upstaging Prediction Model including the presence of PIRADS 5 lesions (AUC = 0.664). Legend: Receiver Operating Characteristic (ROC) curve for the multivariable logistic regression model predicting pathological upstaging, including the presence of PIRADS 5 lesions. The area under the curve (AUC) was 0.664, indicating the model’s modest discriminative ability. The black curve represents the ROC curve of the prediction model, while the red diagonal line indicates no discriminative ability (AUC = 0.5).

Significant risk factors for upstaging were also identified in a subgroup analysis limited to patients with available MRI data (*n* = 326). This model demonstrated the highest predictive accuracy among all analyzed models, with an AUC of 0.664 (Figure 2). PSAD had a strong influence on upstaging (OR 6.102; 95% CI: 2.143–13.374, *p* = 0.001). An increase in PSAD by 0.1 unit resulted in a 19.8% (95% CI: 7.9–33.1) increase in the odds of upstaging. This model also reveals that the detection of PIRADS 5 lesion in mpMRI increases the odds of upstaging by 62% compared to lower PIRADS scores (OR 1.620; 95% CI: 1.014–2.589, *p* = 0.043). Type 2 Diabetes Mellitus (*p* = 0.908), HT (*p* = 0.158), age (*p* = 0.097), and BMI (*p* = 0.942) had no important impact on upstaging in this model (Supplementary Material S2).

### 3.3. MSU

Comparison of patients with and without MSU revealed differences in specific analyzed parameters. Patients with MSU had higher PSA values (14.2 vs. 12.3 ng/mL; *p* < 0.0001) and PSAD (0.40 vs. 0.30 ng/mL/cm^3^; *p* < 0.0001). Significantly smaller prostate volume and a higher rate of HT were also observed in the group with MSU. Moreover, fusion biopsy was performed less frequently in the MSU group (20% vs. 30%; *p* = 0.001). Although fusion biopsy does not directly influence cT classification, it can provide a comprehensive diagnostic overview, as it requires an analysis of mpMRI prior to the biopsy. Thus, it may raise clinicians’ awareness and contribute to a more accurate DRE, thereby improving the accuracy of cT classification.

### 3.4. MSU: Main Model (Entire Cohort)

Multivariable logistic regression analysis, including the entire patient cohort, revealed that higher PSAD was the strongest predictor for MSU (OR 2.02; 95% CI: 1.32–3.10; *p* = 0.001). An increase in PSAD by 0.1 unit resulted in a 7.3% (95% CI: 2.8–12.0%) increase in the odds of MSU. HT (OR 1.54; 95% CI: 1.17–2.03; *p* = 0.002) and fusion biopsy (specifically the lack thereof) (OR 0.58; 95% CI: 0.42–0.81; *p* = 0.001) were also associated with the risk of MSU (Supplementary Material S3). This further confirms the critical role of fusion biopsy in the precise staging of PCa. The presence of upgrading also remained significantly associated with the risk of MSU (OR 1.34; 95% CI: 1.02–1.75; *p* = 0.034). The model’s predictive accuracy was moderate, with an AUC of 0.622.

### 3.5. MSU: Model with PIRADS 5

In the analysis of the subgroup of patients with available MRI imaging data (*n* = 326), risk factors for MSU were also identified. The presence of a PIRADS 5 lesion is associated with higher odds of MSU (OR = 1.75; 95% CI 1.10–2.78; *p* = 0.019). PSAD also remains strongly linked to MSU in this model (OR = 4.67; 95% CI 1.62–13.50; *p* = 0.004). An increase in PSAD by 0.1 unit resulted in a 16.7% (95% CI: 5.0–29.7%) increase in the odds of MSU. In contrast, HT (OR = 1.50; *p* = 0.091) and BMI (OR = 0.90; *p* = 0.092) were not associated with MSU (Supplementary Material S4). The mpMRI-based model demonstrated moderate predictive accuracy, with an AUC of 0.659.

## 4. Discussion

Upstaging and upgrading complicate treatment decisions and outcomes. PCa underestimation leads to a suboptimal care pathway, including incorrect qualification for active surveillance, surgery, and other forms of treatment, as well as the omission of extended lymphadenectomy, inaccurate risk assessment for extracapsular extension (ECE), and seminal vesicle invasion (SVI) [4,8,9]. Moreover, BCR and metastasis-free survival rates are also affected by this phenomenon [4,9].

The discrepancy between biopsy and RP specimens is a known issue that exceeds the sole classification of pathology. Longoni et al. analyzed post-RP outcomes in 9037 patients with favorable intermediate-risk PCa, reporting upstaging in 12.6% and upgrading in 14.8% [10]. By comparison, our analysis revealed a significantly higher upstaging rate of 31.9% in the entire cohort. In most low-risk PCa patients, changes between biopsy and prostatectomy carry limited prognostic impact [11].

Advanced upstaging or upgrading to ISUP ≥ 4 increases cancer-specific mortality and may lead to under-treatment in patients selected for nerve-sparing surgery or limited lymphadenectomy [8,12]. Our study provides valuable insights into the factors associated with pathological upstaging relative to clinical staging (pT vs. cT). In a clinicopathological analysis of 924 patients who underwent RP, pathological upstaging was observed in 31.9% of cases, while MSU occurred in 50.6% of the cohort.

PSAD was the strongest predictor of upstaging and MSU, with ORs of 2.56–6.10 across models. This outcome aligns with previous studies reporting a significant association between higher PSAD values and pathological upstaging and upgrading [13,14,15]. EAU Guidelines consider PSAD > 0.1–0.15 ng/mL/cc as predictive of significant PCa, and <0.09 as low risk [1].

The Prostate Imaging Reporting and Data Sytems (PIRADS) is a well-established MRI-based diagnostic tool. Its substantial diagnostic value of detecting extracapsular extension (ECE) and seminal vesicle invasion (SVI) is extensively documented in the literature [16]. In our study, the presence of a PIRADS 5 lesion was associated with a significant risk of upstaging, a finding that has been previously reported in other research papers [17]. It is worth noting that mpMRI is not always sufficient to rule out advanced PCa, with several studies indicating that a noticeable number of patients with negative mpMRI results develop clinically significant PCa [18,19]. Even though this phenomenon is rare among low-risk groups, the prevalence of high-grade PCs in the patients as mentioned above cannot be ignored.

The role of mpMRI in PCa management, especially for active surveillance, requires further study. In our analysis, the inclusion of PIRADS classification in multivariable models significantly improved their predictive accuracy, with both the upstaging and MSU models showing higher AUC values after the addition of this imaging-based variable.

Our data indicates age as a significant risk factor for upstaging. The population of patients with upstaging was, on average, 1, 4 years older than the population without upstaging. Moreover, logistic regression analysis of the general model showed that each additional year of age corresponded to a 4.2% increase in odds of upstaging, with an AUC of 0.57. The optimal cut-off value, determined using the Youden index, identified 68 years as the threshold for age. Other studies similarly show a 2–3% increase in upstaging risk per year of age [3,20].

Hypertension was identified as an independent predictor in the overall cohort (OR 1.468; 95% CI 1.085–1.986) but not in the MRI subgroup. This topic remains underexplored in the literature. However, some studies suggest that metabolic syndrome is an important component associated with poorer oncologic outcomes in prostate cancer, including more aggressive tumor features, increased risk of extracapsular extension, and high-grade disease. The MRI subgroup, comprising patients who underwent pre-diagnostic MRI, was substantially smaller, which likely limited the ability to detect associations observed in the full cohort due to reduced statistical power. Moreover, this subgroup may represent a more selectively evaluated and potentially earlier-diagnosed population, in which the impact of comorbidities such as hypertension is less pronounced. In addition, the inclusion of MRI-derived variables, such as PIRADS score, may better capture tumor-specific characteristics, thereby attenuating the apparent effect of systemic factors. Consequently, the predictive role of hypertension observed in the overall cohort may be diminished or no longer detectable in the MRI-based model [21,22].

Our study shows that fusion biopsy is associated with a lower risk of MSU (OR = 0.58), highlighting its diagnostic advantage. The observed reduction in inaccurate staging is likely due to several factors. MRI-targeted sampling improves lesion localization and increases detection of clinically significant cancer [23,24]. This provides a more accurate representation of tumor burden than systematic biopsy alone. In addition, pre-biopsy MRI may indirectly refine clinical staging. It can inform digital rectal examination and raise awareness of possible extracapsular extension. However, since our study was retrospective and DRE findings were not standardized, we cannot determine the contribution of these mechanisms. Overall, the improved staging accuracy in our cohort most likely reflects the superior sampling of fusion biopsy, with a potential added benefit from MRI-informed clinical assessment.

PSMA PET/CT improves staging accuracy and nodal detection over mpMRI, though its value in assessing EPE and metastases remains under investigation [25].

The preprostatectomy nomogram developed by Memorial Sloan Kettering Cancer Center (MSKCC) predicts adverse pathology with AUCs of 0.76–0.85, outperforming our models [26]. Similarly, the EAU risk classification uses PSA level, ISUP grade, and cT to stratify patients and guide treatment decisions [1]. Incorporating PIRADS 5 improved model accuracy and may complement MSKCC/EAU classifications in identifying ≥pT3 disease.

This study is subject to several limitations. First, it is a single-center retrospective analysis, which may limit generalizability. Second, although the sample size is substantial, external validation of the predictive models is lacking. Third, the impact of operator variability in mpMRI interpretation and biopsy technique was not assessed. Another limitation of this study is the lack of detailed surgical outcome data, including positive surgical margin rates and nerve-sparing details, especially in relation to PI-RADS findings. As a result, we cannot determine the link between preoperative imaging characteristics and either intraoperative decisions or postoperative oncologic outcomes.

419 patients were excluded because of missing key clinical or pathological data, which may have introduced selection bias and limited the representativeness of the final study cohort. As a result, the analyzed population reflects patients with complete preoperative and pathological information rather than all patients initially screened. Furthermore, MRI data were available only in a subset of 326 patients; therefore, the PIRADS models should be interpreted as subgroup-specific and may have limited generalizability to broader prostate cancer populations.

Finally, genomic or molecular classifiers were not included in this analysis. This matters because tissue-based genomic assays may more precisely identify tumor aggressiveness than standard clinicoradiologic variables. They can improve risk stratification before surgery. Tests such as Decipher and the Genomic Prostate Score (GPS) are linked to adverse pathology at radical prostatectomy for localized prostate cancer [27,28]. These tests may offer more prognostic information than conventional clinical criteria. Integrating them with PSAD, PIRADS findings, and biopsy characteristics could possibly improve our models’ performance (AUC 0.664). This approach may also better identify aggressive, clinically underappreciated disease.

To enhance the clinical relevance of the proposed PSAD thresholds, external validation in an independent cohort is essential. Although the identified cut-off values (0.28–0.29) demonstrated discriminatory ability within the study population, their robustness and generalizability remain uncertain given the single-center, retrospective design. Validation across diverse populations and clinical settings is necessary to confirm reliability, mitigate overfitting risk, and support their integration into routine preoperative risk stratification for upstaging and upgrading.

## 5. Conclusions

Upstaging remains a frequent and clinically relevant phenomenon. This study highlights the persistent challenge of pathological upstaging in PCa. PSAD emerged as the most consistent and robust predictor of upgrading and MSU among evaluated variables. The presence of PIRADS 5 lesions, older age, and HT are also independently associated with an increased risk of upstaging. Simultaneously, performing a fusion biopsy significantly decreases the inaccurate staging. Our findings emphasize the need for comprehensive preoperative risk stratification models that incorporate PSAD and advanced imaging features to improve the accuracy of clinical staging, decreasing the risk of understaging. Although our models showed statistically significant associations, their discriminatory performance remains modest, with the best model reaching an AUC of 0.664, highlighting the need for further optimization and external validation.

## Figures and Tables

**Table 1:** Comparison of Clinical and Demographic Characteristics Between Patients with and without Pathological Upstaging.

Variable	No Upstaging	Upstaging	*p*-Value
Number of patients	629	295	-
Age [years] (mean ± SD)	64.5 ± 6.2	65.9 ± 6.1	**<0.001**
BMI [kg/m^2^] (mean ± SD)	28.0 ± 3.1	27.8 ± 3.0	0.73
Type 2 Diabetes Mellitus (%)	9.1%	9.2%	0.98
Hypertension (%)	44.3%	52.5%	**0.019**
PSA [ng/mL] (mean ± SD)	12.4 ± 12.8	15.1 ± 12.6	**<0.001**
Prostate volume [mL] (mean ± SD)	43.1 ± 19.8	39.5 ± 16.9	**0.004**
PSAD (mean ± SD)	0.319 ± 0.316	0.429 ± 0.392	**<0.001**
Upgrading ISUP (%)	47.8%	54.8%	**0.049**
ISUP 1 (*n* = 452)	346 (76.5%)	106 (23.5%)	
ISUP 2 (*n* = 197)	121 (61.4%)	76 (38.6%)	
ISUP 3 (*n* = 121)	66 (54.5%)	55 (45.5%)	
ISUP 4 (*n* = 88)	60 (68.2%)	28 (31.8%)	
ISUP 5 (*n* = 58)	30 (51.7%)	28 (48.3%)	
ISUP—International Society of Urological Pathology Grade Group, based on preoperative biopsy results. For eight patients, preoperative ISUP data were not available. Continuous variables (age, BMI, PSA, prostate volume, and PSAD) are presented as means ± standard deviations (SD). Percentages refer to the proportion of patients within each subgroup. Upstaging refers to pathological stage ≥ pT3 not suspected preoperatively; No upstaging refers to pathological stage < pT3. Values presented in bold denote statistical significance (*p* < 0.05).			

## Data Availability

The data are available from the authors upon reasonable request.

## Supplementary Materials

The following supporting information can be downloaded at: https://www.mdpi.com/article/10.3390/cancers18081319/s1, Supplementary Material S1: Results of logistic regression of the model including the entire cohort, Supplementary Material S2: Results of logistic regression analy-sis of the model including PIRADS 5, Supplementary Material S3: MSU multivariable logistic regression analysis (entire cohort) including fusion biopsy, Supplementary Material S4: Multivariable logistic regression (PIRADS 5 subgroup).
