# Supplementary material for: PSA Density and PIRADS 5 Lesions as Key Determinants of Upstaging After Radical Prostatectomy
Source: Cancers (Basel). 2026 Apr 21;18(8):1319. doi: 10.3390/cancers18081319 (PMC13114474; doi:10.3390/cancers18081319)
Supplement: Supplementary file 1 [file cancers-18-01319-s001.zip › Supplementary material S1.pdf]

Supplementary Material S1. Results of logistic regression of the model including the entire cohort

Variable	OR (95% CI)	*p-value*
PSAD	2.559 (1.705 – 3.842)	**0.000**
Age during surgery	1.042 (1.017 – 1.067)	**0.001**
Hypertension (HT)	1.468 (1.085 – 1.986)	**0.013**
Type 2 diabetes mellitus (T2DM)	0.885 (0.536 – 1.462)	0.634
BMI	0.971 (0.925 – 1.018)	0.971

*Values in **Table 2** are derived from a logistic regression analysis assessing the risk of pathological upstaging in prostate cancer including the entire cohort.*

- ***OR (Odds Ratio)** – quantifies the strength of association between each variable and the odds of upstaging.*
- ***95% CI (Confidence Interval)** – indicates the range within which the true odds ratio is expected to lie with 95% confidence.*
- ***PSAD** – prostate-specific antigen density, calculated as serum PSA (ng/mL) divided by prostate volume (cm^3).*
- ***Age during surgery** – patient's age at the time of radical prostatectomy.*
- ***Hypertension (HT)** – history of clinically diagnosed arterial hypertension.*
- ***Type 2 diabetes mellitus (T2DM)** – history of clinically diagnosed type 2 diabetes.*
- ***BMI** – body mass index (kg/m^2).*
- *Statistical significance was defined as a p-value < 0.05.*
