# Supplementary material for: PSA Density and PIRADS 5 Lesions as Key Determinants of Upstaging After Radical Prostatectomy
Source: Cancers (Basel). 2026 Apr 21;18(8):1319. doi: 10.3390/cancers18081319 (PMC13114474; doi:10.3390/cancers18081319)
Supplement: Supplementary file 1 [file cancers-18-01319-s001.zip › Supplementary material S2.pdf]

Supplementary Material S2. Results of logistic regression analysis of the model including PIRADS 5

Variable	Odds ratio (OR)	Standard deviation = SD (95% CI)	*p-value*
PSAD	6.102	2.143 - 17.374	**0.001**
Age during surgery	1.032	0.994 - 1.071	0.097
Hypertension (HT)	1.420	0.873 - 2.310	0.158
Type 2 diabetes mellitus (T2DM)	0.958	0.460 - 1.995	0.908
BMI	0.942	0.845 - 1.050	0.942
PIRADS 5	1.620	1.014 - 2.589	**0.043**
BMI	0.942	0.845 - 1.050	0.942

***Table 3** summarizes the results of a multivariable logistic regression model evaluating the association between selected clinical and radiological parameters and the risk of pathological upstaging in patients with available PIRADS 5 data.*

- ***OR (Odds Ratio)** – quantifies the strength of association between each variable and the odds of upstaging.*
- ***95% CI (Confidence Interval)** – indicates the range within which the true odds ratio is expected to lie with 95% confidence.*
- ***PIRADS 5** – the presence of a lesion categorized as score five on multiparametric MRI, based on the Prostate Imaging–Reporting and Data System (PIRADS) version 2.1.*
- ***PSAD** – prostate-specific antigen density, calculated as serum PSA (ng/mL) divided by prostate volume (cm^3).*
- ***Age during surgery** – patient's age at the time of radical prostatectomy.*
- ***Hypertension (HT)** – history of clinically diagnosed arterial hypertension.*
- ***Type 2 diabetes mellitus (T2DM)** – history of clinically diagnosed type 2 diabetes.*
- ***BMI** – body mass index (kg/m^2).*
- *Statistical significance was defined as a p-value < 0.05.*
