# Supplementary material for: PSA Density and PIRADS 5 Lesions as Key Determinants of Upstaging After Radical Prostatectomy
Source: Cancers (Basel). 2026 Apr 21;18(8):1319. doi: 10.3390/cancers18081319 (PMC13114474; doi:10.3390/cancers18081319)
Supplement: Supplementary file 1 [file cancers-18-01319-s001.zip › Supplementary material S3.pdf]

Supplementary Material S3. MSU multivariable logistic regression analysis (entire cohort) including fusion biopsy

Variable	OR (95% CI)	*p-value*
PSAD	2.02 (1.32–3.10)	**0.001**
HT	1.54 (1.17–2.03)	**0.002**
Fusion biopsy	0.58 (0.42–0.81)	**0.001**
Upgrading	1.34 (1.02–1.75)	**0.034**

***Table 4** presents the results of a multivariable logistic regression analysis assessing predictors of pathological upstaging in prostate cancer including fusion biopsy.*

- ***OR (Odds Ratio)** – quantifies the strength of association between each variable and the odds of upstaging.*
- ***95% CI (Confidence Interval)** – indicates the range within which the true odds ratio is expected to lie with 95% confidence.*
- ***PSAD** – prostate-specific antigen density, calculated as serum PSA (ng/mL) divided by prostate volume (cm^3).*
- ***Hypertension (HT)** – history of clinically diagnosed arterial hypertension.*
- *Fusion biopsy – use of MRI–TRUS fusion-guided biopsy technique (reference: standard biopsy).*
- *Upgrading – the presence of pathological upgrading (higher ISUP grade group in final histopathology compared to biopsy).*
- *Statistical significance was defined as a p-value < 0.05.*
