# Supplementary material for: PSA Density and PIRADS 5 Lesions as Key Determinants of Upstaging After Radical Prostatectomy
Source: Cancers (Basel). 2026 Apr 21;18(8):1319. doi: 10.3390/cancers18081319 (PMC13114474; doi:10.3390/cancers18081319)
Supplement: Supplementary file 1 [file cancers-18-01319-s001.zip › Supplementary material S4.pdf]

Supplementary Material S4. Multivariable logistic regression (PIRADS 5 subgroup)

Variables	OR (95% CI)	*p*-value
PSAD	4.67 (1.62–13.50)	**0.004**
PIRADS 5	1.75 (1.10–2.78)	**0.019**
HT	1.50 (0.94–2.38)	0.091
BMI	0.90 (0.80–1.02)	0.092

Table 5 *presents the results of a multivariable logistic regression model evaluating predictors of pathological upstaging in the subgroup of patients with PIRADS 5 lesions on multiparametric MRI.*

- ***PSAD*** *– prostate-specific antigen density, calculated as serum PSA (ng/mL) divided by prostate volume (cm^3).*
- ***PIRADS 5*** *– the presence of a lesion categorized as a score 5 on multiparametric MRI, based on the Prostate Imaging–Reporting and Data System (PIRADS) version 2.1.*
- ***Hypertension (HT)*** *– history of clinically diagnosed arterial hypertension.*
- ***BMI*** *– body mass index (kg/m^2).*
